# Aggregated Recommendation through Random Forests

**DOI:** 10.1155/2014/649596

**Published:** 2014-08-11

**Authors:** Heng-Ru Zhang, Fan Min, Xu He

**Affiliations:** ^1^School of Computer Science, Southwest Petroleum University, Chengdu 610500, China; ^2^Lab of Granular Computing, Minnan Normal University, Zhangzhou 363000, China

## Abstract

Aggregated recommendation refers to the process of suggesting one kind of items to a group of users. Compared to user-oriented or item-oriented approaches, it is more general and, therefore, more appropriate for cold-start recommendation. In this paper, we propose a random forest approach to create aggregated recommender systems. The approach is used to predict the rating of a group of users to a kind of items. In the preprocessing stage, we merge user, item, and rating information to construct an aggregated decision table, where rating information serves as the decision attribute. We also model the data conversion process corresponding to the new user, new item, and both new problems. In the training stage, a forest is built for the aggregated training set, where each leaf is assigned a distribution of discrete rating. In the testing stage, we present four predicting approaches to compute evaluation values based on the distribution of each tree. Experiments results on the well-known MovieLens dataset show that the aggregated approach maintains an acceptable level of accuracy.

## 1. Introduction

Recommender systems (RSs) [1–3] have been extensively studied to present items, such as movies, music, and books. They collect information on the preferences of their users for a set of items. The information is used to fulfill two main user tasks; one is predicting the rating [4], and the other is finding good items [5].

Model-based RSs apply demographic or content information to construct a model. Some algorithms, such as Bayesian classifiers [6] and decision trees [7], have been used to generate respective models. Model-based algorithms are suitable for cold-start recommendation for new item, new user, and new community [2, 8, 9].

Aggregated RS aims at recommending a kind of items to a group of users. A popular approach is to jointly recommend items to user groups [10] (e.g., a group of four friends who wish to choose a movie). Given the specific characteristics of the recommendation to groups, Jameson and Smyth [11] proposed to appropriately establish a consensus for different group semantics that formalize the agreements and disagreements among users.

In this paper, we propose a random forest (RF) approach to create aggregated RS through taking advantage of demographic, content, and rating information. This approach intends to predict the ratings of a group of users to a kind of items and deal with three cold-start recommendation problems, namely, new item (NI), new user (NU), and double cold-start (DOCS), where new items are recommended to new users. Decision tree [12] is a natural approach to these problems. However, one decision tree only takes advantage of limited information of users and items. Therefore, for many new users and new items, a decision tree may produce no predicting result at all. Our approach uses different information of users and items to construct an RF (a collection of decision trees [13]) to avoid this situation.

Our approach has three stages. In the preprocessing stage, the user, item, and rating tables of the original dataset are merged into an aggregated table. Then we construct training and testing sets through cross-validation. To the best of the authors' knowledge, little is known regarding the aggregated approach. In the training stage, an RF predictor is built to ensemble individual tree predictors. Each decision tree classifier is generated from the training set with each leaf assigned a distribution of the class attribute. In the testing stage, the demographic and content information of each user-item pair are fed to all decision trees in the RF. Each instance can get the class distribution information through top-down search. We adopt four predicting approaches, called standard voting, weighted average, distribution aggregation based voting, and distribution aggregation based average, to compute evaluation values of the RF.

The contribution of the paper is fourfold. First, we propose a new aggregated approach to predict the rating of a group of users to a kind of items. Second, we merge demographic, content, and rating information into a new aggregated table and then adopt three kinds of strategies, namely, NI, NU, and DOCS, to split training and testing sets through cross-validation. Third, we build five kinds of aggregated RSs by RF. The first three RFs are NI, NU, and DOCS. The two other RFs are new item with average rating (NIAR) and new user with average rating (NUAR). DOCS RS can be used to recommend a kind of new items to a group of new users. Fourth, we develop four ensemble approaches to compute predicting ratings of these aggregated RSs.

Experiments are undertaken with five scenarios corresponding to five aggregated RSs. The abovementioned ensemble approaches are employed to find the appropriate setting of the forest size and compare the performance with respect to the mean absolute error (MAE) [14] in each scenario. MAE is a statistical accuracy metric that measures the deviation between real ratings and predictions generated by the RS. If {*r*_1_,…, *r*_*N*_} are all the real values in the target set, {*p*_1_,…, *p*_*N*_} are the predicted values for the same ratings, and *E* = {*ε*_1_,…, *ε*_*N*_} = {*p*_1_ − *r*_1_,…, *p*_*N*_ − *r*_*N*_}, then the MAE is

(1) $$\begin{array}{c} \;\left|\bar{E}\right|=\frac{\Sigma_{i=1}^{N}\left(\left|\varepsilon_{i}\right|\right)}{N}. \end{array}$$

The lower the MAE, the more accurate the approach.

Experimental results on the well-known MovieLens dataset show that (1) the size of the forest is not large to ensure that the performance in terms of MAE keeps stable; (2) the aggregated approach maintains an acceptable level of accuracy.

## 2. Data Models

In this section, the original data sets are converted to an aggregated decision table. Five kinds of aggregated data models are constructed through cross-validation.

### 2.1. Information Systems and Decision Systems

In this subsection, we revisit the definitions of information system [15] and decision systems.

**Definition 1 .**

*S* = (*U*, *A*) is an information system, where *U* = {*x*_1_, *x*_2_,…, *x*_*n*_} is the set of all objects, *A* = {*a*_1_, *a*_2_,…, *a*_*m*_} is the set of all attributes, and *a*_*j*_(*x*_*i*_) is the value of *x*_*i*_ on attribute *a*_*j*_ for *i* ∈ [1,…, *n*] and *j* ∈ [1,…, *m*].

**Example 2 .**

**Figure 1:**
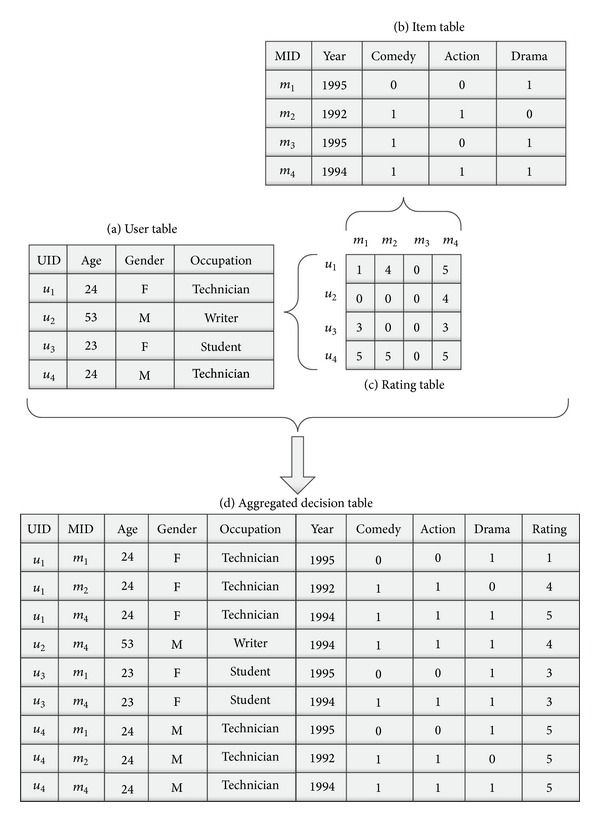
Aggregated decision system.

An example of information system is given by Table (a) of Figure 1, where *U* = {*u*_1_, *u*_2_, *u*_3_, *u*_4_} and *A* = {UID, Age, Gender, Occupation}. UID is a key. Another example of information system is given by Table (b) of Figure 1.

The decision system is a fundamental concept in data mining and machine learning and is often defined as follows [16].

**Definition 3 .**

A decision system *S* is the 5-tuple:

(2) $$\begin{array}{c} S=\left(U,C,D,\left\{V_{a}\;|\;a\in C\cup D\right\},\left\{I_{a}\;|\;a\in C\cup D\right\}\right), \end{array}$$

where *U* is a finite set of objects called the universe, *C* is the set of conditional attributes, *D* is the set of decision attributes, *V*_*a*_ is the set of values for each *a* ∈ *C* ∪ *D*, and *I*_*a*_∣*U* → *V*_*a*_ is an information function for each *a* ∈ *C* ∪ *D*.

### 2.2. Rating System

In this subsection, the rating system is defined.

**Definition 4 .**

Let *U* = {*x*_1_, *x*_2_,…, *x*_*n*_} and *V* = {*y*_1_, *y*_2_,…, *y*_*l*_} be two sets of objects.

Consider

(3) $$\begin{array}{c} R:U\times V⟶R_{k}, \end{array}$$

where *R*_*k*_ is the domain of rating.

If *R*_*k*_ is boolean, *R* is a binary relation from *U* to *V*. If *R*_*k*_ is numeric, *R* is a rating function from *U* to *V*. In this paper, we discuss the numeric ratings. A rating function is more often stored in the database as a table with two foreign keys. In this way the storage is saved. For the convenience of illustration, here we represented it with an *n* × *l* rating matrix.

With Definitions 1 and 4, we propose the following definition.

**Definition 5 .**

A rating system is a 5-tuple RS = (*U*, *A*, *V*, *B*, *R*), where (*U*, *A*) and (*V*, *B*) are two information systems, and *R* : *U* × *V* → *R*_*k*_ is a rating function.

**Example 6 .**

An example of rating is given by Table (c) of Figure 1, where *U* is the set of users as indicated by Table (a) of Figure 1 and *V* is the set of items as indicated by Table (b) of Figure 1. Here *R*_*k*_ = {0,1, 2,3, 4,5}.

An example of rating system includes Tables (a), (b), and (c) of Figure 1.

### 2.3. Aggregated Decision Systems

In this subsection, we build decision systems to mine the behavior of users on items. For this purpose, we propose the concept of aggregated decision system as follows.

**Definition 7 .**

An aggregated decision system induced by a rating system (RS) is

(4) $$\begin{array}{c} \text{DS}\left(\text{RS}\right)=\left(G,C,d\right), \end{array}$$

where *G* = *U* × *V* and *C* = *A* ∪ *B*, for all *g*_*i*,*j*_ = (*x*_*i*_, *y*_*j*_) ∈ *G*, *d*(*g*_*i*,*j*_) = *R*(*x*_*i*_, *y*_*j*_).

The number of objects in DS(RS) is |*U*| × |*V*|. To distinguish this type of decision system from the other types discussed later, we refer to it as the* aggregated decision system* (ADS) or the* first*-*class decision system* (1-DS).

In Table (c) of Figure 1 some elements are 0, in which zero element means that a user has not rated the movie. We remove them and get a new decision system DS^+^(RS) as follows.

**Definition 8 .**

An aggregated decision system with positive rating induced by a rating system (RS) is

(5) $$\begin{array}{c} \text{D}{\text{S}}^{+}\left(\text{RS}\right)=\left(G^{+},C,d\right), \end{array}$$

where *G* ^+^ = {(*x*, *y*)∣*x* ∈ *U*, *y* ∈ *V*, *R*(*x*, *y*) > 0} and *C* = *A* ∪ *B*, ∀*g*_*i*,*j*_ = (*x*_*i*_, *y*_*j*_) ∈ *G* ^+^, *d*(*g*_*i*,*j*_) = *R*(*x*_*i*_, *y*_*j*_).

The number of objects in DS^+^(RS) is |*G* ^+^|. We refer to it as the ADS^+^ or the* second*-*class decision system* (2-DS).

**Example 9 .**

Table (d) of Figure 1 presents a ADS^+^ decision system where *G* ^+^ = {(*u*_1_, *m*_1_), (*u*_1_, *m*_2_), (*u*_1_, *m*_4_), (*u*_2_, *m*_4_), (*u*_3_, *m*_1_), (*u*_3_, *m*_4_), (*u*_4_, *m*_1_), (*u*_4_, *m*_2_), (*u*_4_, *m*_4_)}, *C* = {Age, Gender,  Occupation, Year, Comedy, Action, Drama}, and *d* = {Rating}. (UID, MID) is a key pair. The key pair does not participate in the establishment of RF model; therefore, it is not used in the mining work.

The attribute of average rating (AR) has been the focus of most empirical studies on product reviews [17]. There are two kinds of AR. One kind is AR of user (UAR), which reflects the rating habit for the user. The other is AR for item (IAR), which reflects the degree of item popularity. With Definition 8, we can define a new type of the aggregated decision system with AR as follows.

**Definition 10 .**

An aggregated decision system with AR(RDS^+^) or the* third-class decision system* (3-DS) is

(6) $$\begin{array}{c} \text{D}{\text{S}}_{\text{AR}}^{+}\left(\text{RS}\right)=\left(G^{+},C^{\prime },d\right), \end{array}$$

where *G* ^+^ = {(*x*, *y*)∣*x* ∈ *U*, *y* ∈ *V*, *R*(*x*, *y*) > 0} and *C*′ = *A* ∪ *B* ∪ {AR}, ∀*g*_*i*,*j*_ = (*x*_*i*_, *y*_*j*_) ∈ *G*, *d*(*g*_*i*,*j*_) = *R*(*x*_*i*_, *y*_*j*_).

**Example 11 .**

The movies *m*_1_, *m*_2_, and *m*_4_ are rated by user *u*_1_ in Table (c) of Figure 1. Therefore, the AR of *u*_1_ is ⌊(1 + 4 + 5)/3⌋ = 3. Similarly, the AR of *u*_4_ is ⌊(5 + 5 + 5)/3⌋ = 5.

The movie *m*_1_ is rated by the users *u*_1_, *u*_3_, and *u*_4_ in Table (c) of Figure 1. Therefore, the AR for *m*_1_ is ⌊(1 + 3 + 5)/3⌋ = 3. Similarly, the AR for *m*_4_ is ⌊(5 + 4 + 3 + 5)/4⌋ = 4.

In some situations we are interested in the aggregated decision system concerning a subset of users and items.

**Definition 12 .**

A subset of the aggregated decision system (SDS^+^) or the* fourth*-*class decision system* (4-DS) with respect to *U*′ and *V*′ is

(7) $$\begin{array}{c} \text{D}{\text{S}}^{+}\left(\text{RS},U^{\prime },V^{\prime }\right)=\left(G^{\prime },C,d\right), \end{array}$$

where *G*′ = {(*x*, *y*)∣*x* ∈ *U*′, *y* ∈ *V*′, *R*(*x*, *y*) > 0}, *U*′⊆*U*, *V*′⊆*V*, and *C* = *A* ∪ *B*, ∀*g*_*i*,*j*_ = (*x*_*i*_, *y*_*j*_) ∈ *G*′, *d*(*g*_*i*,*j*_) = *R*(*x*_*i*_, *y*_*j*_).

**Definition 13 .**

A subset of the aggregated decision system with AR (SRDS^+^) or the* fifth-class decision system* (5-DS) with respect to *U*′ and *V*′ is

(8) $$\begin{array}{c} \text{D}{\text{S}}_{\text{AR}}^{+}\left(\text{RS},U^{\prime },V^{\prime }\right)=\left(G^{\prime },C^{\prime },d\right), \end{array}$$

where *G*′ = {(*x*, *y*)∣*x* ∈ *U*′, *y* ∈ *V*′, *R*(*x*, *y*) > 0}, *U*′⊆*U*, *V*′⊆*V*, and *C*′ = *A* ∪ *B* ∪ AR, ∀*g*_*i*,*j*_ = (*x*_*i*_, *y*_*j*_) ∈ *G*′, *d*(*g*_*i*,*j*_) = *R*(*x*_*i*_, *y*_*j*_).

In this paper, we discuss the cold-start problem. In Definitions 12 and 13, the demographic or content information of the subsets is not independent. Therefore, the subsets are not used to solve cold-start problem.

### 2.4. Data Splitting

For proper estimation of the classification accuracy, the decision sytem is divided into training and testing sets. The training set is used to calculate a classifier, which is used to classify the testing set.

We adopt three kinds of splitting strategies based on ADS^+^, namely, NU, NI, and DOCS. Two kinds of splitting strategies are adopted based on RDS^+^, namely, NUAR and NIAR. NU and NUAR approaches split the user table into two parts. Then each part is merged into item and rating information, respectively, to construct training and testing sets. A sample is that the number of training or testing sets is 1/2 of the original set. NI and NIAR approaches split the item table into two parts. Then each part is merged into user and rating information, respectively, to construct training and testing sets. A sample is that the number of training or testing sets is 1/2 of the original set.

DOCS approach splits user table and item table into two parts, respectively. The first part of user table, the first part of item table, and rating information are merged into training set. And the second part of user table, the second part of item table, and rating information are merged into testing set. A sample is that the number of training or testing sets is 1/4 of the original set.

Supposing a group of new users and the item model, the function predicts whether these users would be interested in a set of items. With Definitions 8 and 12, the training or testing set of NU is defined as follows.

**Definition 14 .**

A subset of the aggregated decision system with respect to user sampling NU is

(9) $$\begin{array}{c} \text{D}{\text{S}}_{\text{NU}}^{+}\left(\text{RS},U^{\prime },V\right)=\left(G^{\prime },C,d\right), \end{array}$$

where *U*′⊆*U*, *G*′ = {(*x*, *y*)∣*x* ∈ *U*′, *y* ∈ *V*, *R*(*x*, *y*) > 0}.

Give *U*_*r*_′ ≠ *∅* and *U*_*t*_′ ≠ *∅*, where *U*_*r*_′ ∪ *U*_*t*_′ = *U* and *U*_*r*_′∩*U*_*t*_′ = *∅*. While *U*′ = *U*_*r*_′, DS_NU_^+^(RS, *U*′, *V*) is the training set. While *U*′ = *U*_*t*_′, DS_NU_^+^(RS, *U*′, *V*) is the testing set.

Given a set of new items and the user model, the function predicts whether a group of users would be interested in these items. With Definitions 8 and 12, the training or testing set of NI is defined as follows.

**Definition 15 .**

A subset of the aggregated decision system with respect to user sampling NI is

(10) $$\begin{array}{c} \text{D}{\text{S}}_{\text{NI}}^{+}\left(\text{RS},U,V^{\prime }\right)=\left(G^{\prime },C,d\right), \end{array}$$

where *V*′⊆*V*, *G*′ = {(*x*, *y*)∣*x* ∈ *U*, *y* ∈ *V*′, *R*(*x*, *y*) > 0}.

Give *V*_*r*_′ ≠ *∅* and *V*_*t*_′ ≠ *∅*, where *V*_*r*_′ ∪ *V*_*t*_′ = *V* and *V*_*r*_′∩*V*_*t*_′ = *∅*. While *V*′ = *V*_*r*_′, DS_NI_^+^(RS, *U*, *V*′) is the training set. While *V*′ = *V*_*t*_′, DS_NI_^+^(RS, *U*, *V*′) is the testing set.

With Definitions 12, 14, and 15, the training or testing set of DOCS is defined as follows.

**Definition 16 .**

A subset of the aggregated decision system with respect to user sampling DOCS is

(11) $$\begin{array}{c} \text{D}{\text{S}}_{\text{DOCS}}^{+}\left(\text{RS},U^{\prime },V^{\prime }\right)=\left(G^{\prime },C,d\right), \end{array}$$

where *U*′⊆*U*, *V*′⊆*V*, and *G*′ = {(*x*, *y*)∣*x* ∈ *U*′, *y* ∈ *V*′, *R*(*x*, *y*) > 0}.

Give *U*_*r*_′ ≠ *∅* and *U*_*t*_′ ≠ *∅*, where *U*_*r*_′ ∪ *U*_*t*_′ = *U* and *U*_*r*_′∩*U*_*t*_′ = *∅*. Give *V*_*r*_′ ≠ *∅* and *V*_*t*_′ ≠ *∅*, where *V*_*r*_′ ∪ *V*_*t*_′ = *V* and *V*_*r*_′∩*V*_*t*_′ = *∅*. While *U*′ = *U*_*r*_′ and *V*′ = *V*_*r*_′, DS_DOCS_^+^(RS, *U*′, *V*′) is the training set. While *U*′ = *U*_*t*_′ and *V*′ = *V*_*t*_′, DS_DOCS_^+^(RS, *U*′, *V*′) is the testing set.

With Definitions 10 and 13, the training or testing set of NUAR is defined as follows.

**Definition 17 .**

A subset of the aggregated decision system with respect to user sampling NUAR is

(12) $$\begin{array}{c} \text{D}{\text{S}}_{\text{NUAR}}^{+}\left(\text{RS},U^{\prime },V\right)=\left(G^{\prime },C^{\prime },d\right), \end{array}$$

where *U*′⊆*U*, *G*′ = {(*x*, *y*)∣*x* ∈ *U*′, *y* ∈ *V*, *R*(*x*, *y*) > 0}, and *C*′ = *A* ∪ *B* ∪ {*IAR*}.

Let *U*_*r*_′ ∪ *U*_*t*_′ = *U*, *U*_*r*_′∩*U*_*t*_′ = *∅*, *U*_*r*_′ ≠ *∅*, and *U*_*t*_′ ≠ *∅*. While *U*′ = *U*_*r*_′, DS_NUAR_^+^(RS, *U*′, *V*) is the training set. While *U*′ = *U*_*t*_′, DS_NUAR_^+^(RS, *U*′, *V*) is the testing set.

With Definitions 10 and 13, the training or testing set of NIAR is defined as follows.

**Definition 18 .**

A subset of the aggregated decision system with respect to user sampling NIAR is

(13) $$\begin{array}{c} \text{D}{\text{S}}_{\text{NIAR}}^{+}\left(\text{RS},U,V^{\prime }\right)=\left(G^{\prime },C^{\prime },d\right), \end{array}$$

where *V*′⊆*V*, *G*′ = {(*x*, *y*)∣*x* ∈ *U*, *y* ∈ *V*′, *R*(*x*, *y*) > 0}, and *C*′ = *A* ∪ *B* ∪ {*UAR*}.

Let *V*_*r*_′ ∪ *V*_*t*_′ = *V*, *V*_*r*_′∩*V*_*t*_′ = *∅*, *V*_*r*_′ ≠ *∅*, and *V*_*t*_′ ≠ *∅*. While *V*′ = *V*_*r*_′, DS_NIAR_^+^(RS, *U*, *V*′) is the training set. While *V*′ = *V*_*t*_′, DS_NIAR_^+^(RS, *U*, *V*′) is the testing set.

## 3. Random Forest Based Prediction

In this section, an RF for aggregated dataset is constructed. Four kinds of predicting approaches will be used to compute evaluation values.

### 3.1. Construct the Random Tree

There are two aspects to build an RF: (1) random decision trees are built based on the aggregated training set; (2) an RF is constructed based on these trees.

Decision tree learners build a decision tree by recursively partitioning training data. In the build process of random decision tree, demographic and content information serve as conditional attributes, and rating information serves as the decision attribute. Each root-to-leaf path of tree represents a rule for the ratings of one kind of movies by one group of users.

**Example 19 .**

In Table (d) of Figure 1, the conditional attributes are {Age, Gender, Occupation, Year, Comedy, Action, Drama} and the decision attribute is {Rating}.

There are four steps to build random decision tree: (1) an attribute is randomly selected from the conditional ones as the root node, when the information gain of the attribute is more than 0; (2) the original set will be split to many subsets based on values of the root node; (3) other splitting nodes are constructed based on algorithm of random decision tree, and these subsets will be split recursively to construct subtrees; (4) the leaves are assigned the vector which indicates the distribution of the decision values. The building process of a random decision tree is described in Algorithm 1.

The following examples illustrate the selection process of root node and the way to get the distribution of leaf node.

**Example 20 .**

**Figure 2:**
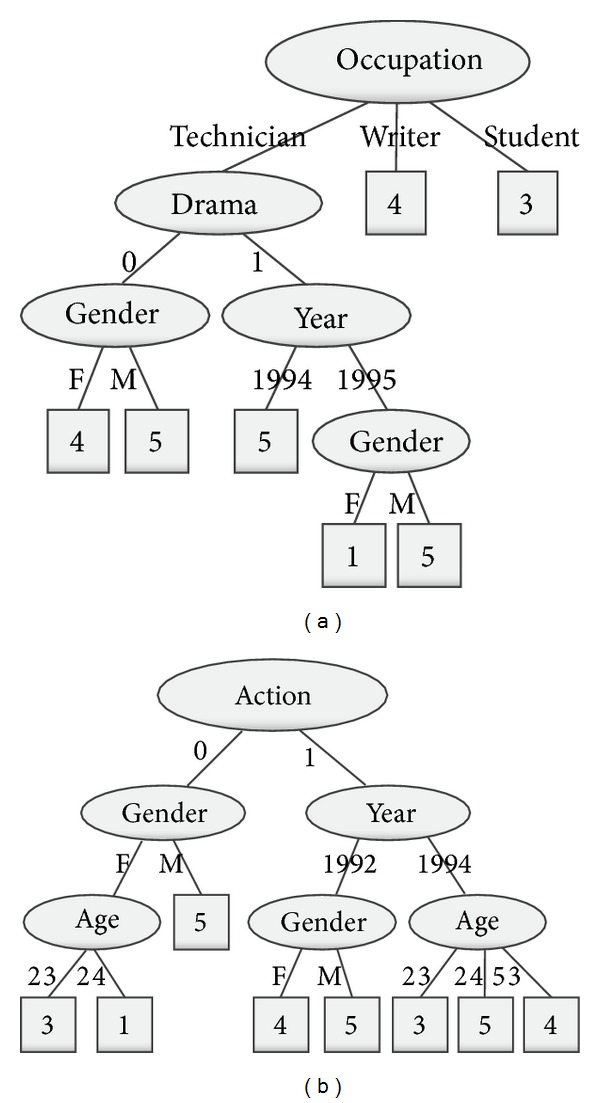
Samples of random decision tree.

The conditional attribute is randomly selected as tree node. After a randomized selection, the root node of Figure 2(a) is {Occupation}, and the root node of Figure 2(b) is {Action}. Then we illustrate the subtree and leaf-node construction process of Figure 2(a). The training data is split according to three values {technician}, {writer}, and {student} of {Occupation}. {Drama} is randomly selected as subtree node corresponding to the value {technician}. The leaf node corresponding to the value {student} is the distribution of the decision values. There are two instances corresponding to the value {student}, and the decision value of the two instances is the same rating based on the aggregated decision table of Figure 1. Therefore, the distribution of the leaf node is {0, 0, 0, 2, 0, 0} corresponding to the rating rated from {student}. If standard voting to the distribution is used when the leaf node is built, the leaf of {student} is 3. In other words, the root-to-leaf path of the tree represents a rule that the rating of all movies rated by the* student* is 3.

A random decision tree only takes advantage of limited information of users and items. Therefore, for many new users and new items, a random decision tree may produce no predicting result at all. For example, the tree of Figure 2(a) has no {Age} information of users. No predicting result is produced if based on the classifier of {Age} information. But two trees of Figures 2(a) and 2(b) can avoid this situation.

### 3.2. Construct the Forest

Based on five kinds of splitting approaches mentioned in Section 2.4, we construct five kinds of RFs: (1) NU forest, (2) NUAR forest, (3) NI forest, (4) NIAR forest, and (5) DOCS forest. NU and NUAR forests are only used to solve new user problem. The two models can predict the rating of a group of new users to a kind of items. NI and NIAR forests are only used to solve new item problem. The two models can predict the rating of a group of users to a kind of new items. DOCS forest can be used to solve both new user and item problems. The model can predict the rating of a group of new users to a kind of new items.

There are three steps to build the RF. (1) Aggregated training and testing set are generated according to the different RF models. For NU forest, the original data is split based on Definition 14. For NUAR forest, the original data is split based on Definition 17. For NI forest, the original data is split based on Definition 15. For NIAR forest, the original data is split based on Definition 18. For DOCS forest, the original data is split based on Definition 16. (2) Condition attributes are randomized based on random seed. (3) Build *N* random decision trees based on Algorithm 1. *N* designated by the user is the size of forest. The building process of the random forest is described in Algorithm 2.

**Figure 3:**
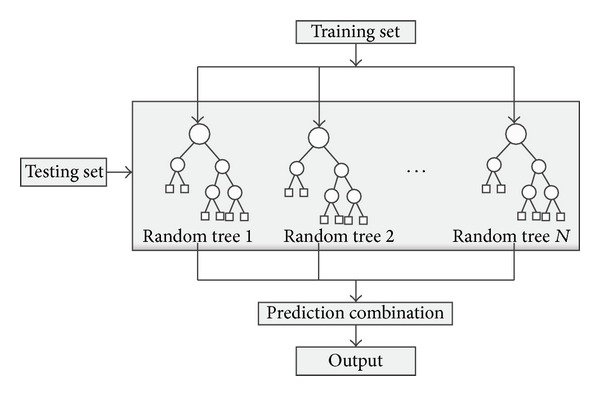
Random forest based prediction.

After the RFs are built, we can use them to predict.  Figure 3 depicts the RF's building and predicting process. Multiple RFs are built through selecting different numbers of random trees. Each tree in Figure 3 uses a different random seed; therefore, each one significantly contributes to the prediction.

### 3.3. Predicting Approaches

For each RF, we design four prediction approaches: (1) standard voting, (2) weighted average, (3) distribution aggregation based voting, and (4) distribution aggregation based average.

By comparing approaches (1) and (2), we can know which is more precise between standard voting and weighted average. By comparing approaches (3) and (4), we can know which is more precise between distribution aggregation based voting and distribution aggregation based average. By comparing approaches (1) and (3), we can know which is more precise between standard voting and distribution aggregation based voting. By comparing approaches (2) and (4), we can know which is more precise between weighted average and distribution aggregation based average.

We describe the four combination algorithms as follows.

*(1) Standard Voting.* For each instance of testing set, there are three steps to get the predicting rating through standard voting. First, each predicting rating is computed in each decision tree of RF through standard voting. These predicting ratings are discrete value. Second, the number of random trees is counted corresponding to each predicting rating. Third, the rating supported by the largest population of trees is used as the RF predicting value. This is given by

(14) $$\begin{array}{c} p=r_{v}^{\max}, \end{array}$$

where *v* is count of random trees.

The following example illustrates the three steps.

*(2) Weighted Average.* For each instance of testing set, there are two steps through weighted average. The first step is the same as the first one of standard voting. Second, the weighted average of these predicting ratings is computed as the RF predicting value. This is given by

(15) $$\begin{array}{c} p=\frac{\Sigma_{i=1}^{r_{\max}}i*\left(\ln\left(v_{i}\right)\right)}{\Sigma_{i=1}^{r_{\max}}\left(\ln\left(v_{i}\right)\right)}, \end{array}$$

where *r*_max⁡_ is the highest rating.

The following example illustrates the two steps.

**Example 21 .**

Based on Example 20 and Figure 2(a), the distribution of the leaf node is {0, 0, 0, 2, 0, 0} corresponding to the value of {student} if *r*_max⁡_ is 5. When an instance of testing set gets the distribution after traversing the random decision tree classifier, the predicting rating of the instance is 3 through standard voting.

Assume there are 10 trees of an RF. The number of random trees is 5 corresponding to the predicting rating 4. The number of random trees is 3 corresponding to the predicting rating 2. The number of random trees is 2 corresponding to the predicting rating 3.

The final predicting value of standard voting is 4, and then the final predicted value of weighted average is given by

(16) $$\begin{array}{c} p=\left\lfloor \frac{\left(5*\ln\left(4\right)+2*\ln\left(3\right)+3*\ln\left(2\right)\right)}{\left(\ln\left(4\right)+\ln\left(3\right)+\ln\left(2\right)\right)}\right\rfloor =5. \end{array}$$

*(3) Distribution Aggregation Based Voting.* For each instance of testing set, there are three steps to get the predicting rating through distribution aggregation based voting. First, each predicting distribution is obtained through top-down search of each decision tree of RF. Second, the cumulative vector is computed through accumulating the predicted distributions of all trees. Third, the final predicted value of the RF is computed based on the cumulative vector through standard voting.

*(4) Distribution Aggregation Based Average.* For each instance of testing set, there are three steps to get the predicting rating through distribution aggregation based average. The previous two steps of the algorithm are the same as distribution aggregation based voting. The third step is different between two algorithms. The final predicted value of the RF is computed based on the cumulative vector through average.

**Example 22 .**

Assume there are 3 trees of an RF. The predicted distribution of the first tree is {0, 0, 0, 3, 0, 0}. The predicted distribution of the second tree is {0, 0, 0, 1, 0, 4}. The predicted class distribution of the third tree is {0, 0, 0, 3, 1, 0}. The cumulative vector is {0, 0, 0, 7, 1, 4}.

The final predicted value of distribution aggregation based voting is 3, and then the final predicting value of distribution aggregation based average is given by

(17) $$\begin{array}{c} p=\left\lfloor \frac{\left(3*7+4*1+5*4\right)}{\left(7+1+4\right)}\right\rfloor =4. \end{array}$$

## 4. Experimental Results

In Section 3, we have designed five kinds of RFs and each kind has four predicting approaches. In this section, we finish a total of 20 forecast schemes. Each scheme is repeated 10 times with different random partitions into training and testing sets (i.e., 10 × cross-validation).

We try to answer the following questions through experimentation.

1. How large is the size of RF when the precision in terms of MAE keeps stable?
2. Which is more precise, in terms of MAE, NU, NI, or DOCS?
3. Which is more precise, in terms of MAE, NU or NUAR?
4. Which is more precise, in terms of MAE, NI or NIAR?
5. Which is more precise, in terms of MAE, standard voting, weighted average, distribution aggregation based voting, or distribution aggregation based average?

### 4.1. Dataset

We experimented with a well-known MovieLens dataset (http://www.movielens.org/) assembled by the GroupLens project. It is widely used in recommender systems (see, e.g., [18]). The database schema is as follows:

- user (userID, age, gender, and occupation),
- movie (movieID, release-year, and genre),
- rates (userID and movieID).

We use the version with 943 users and 1,682 movies. The original rate relation contains the rating of movies with 5 scales. The user age has 61 attributes as indicated by the data set. The user occupation has 21 attributes. Since there are 85 attributes of the movie release-year, the genre is a multivalued attribute. Therefore, we scale it to 18 boolean attributes, namely, action, adventure, animation, children, comedy, crime, documentary, drama, fantasy, FilmNoir, horror, musical, mystery, romance, scientific-fiction, thriller, war, and western. All users have watched at least one movie, and the dataset consists of approximately 100,000 movies ratings. But rating matrix is still spare because no one has watched more than 45 percent of the total movies, and only the 20 percent users have watched more than 10 percent movies.

### 4.2. Results

The original dataset is partitioned into training set and testing set through cross-validation. The training set is 80% of the original one, and the testing set is 20% of the original one.

In order to know the size of the forest when the precision in terms of MAE keeps stable, the number of random trees defined by us is from 2 to 50. We undertake 20 sets of experiments to answer the questions raised at the beginning of the section one by one. Each experiment is repeated 10 times with different sampling of training and testing sets, and the average accuracy in terms of MAE [19] is computed. MAE has been used to evaluate recommender systems in several cases [20, 21].

**Figure 4:**
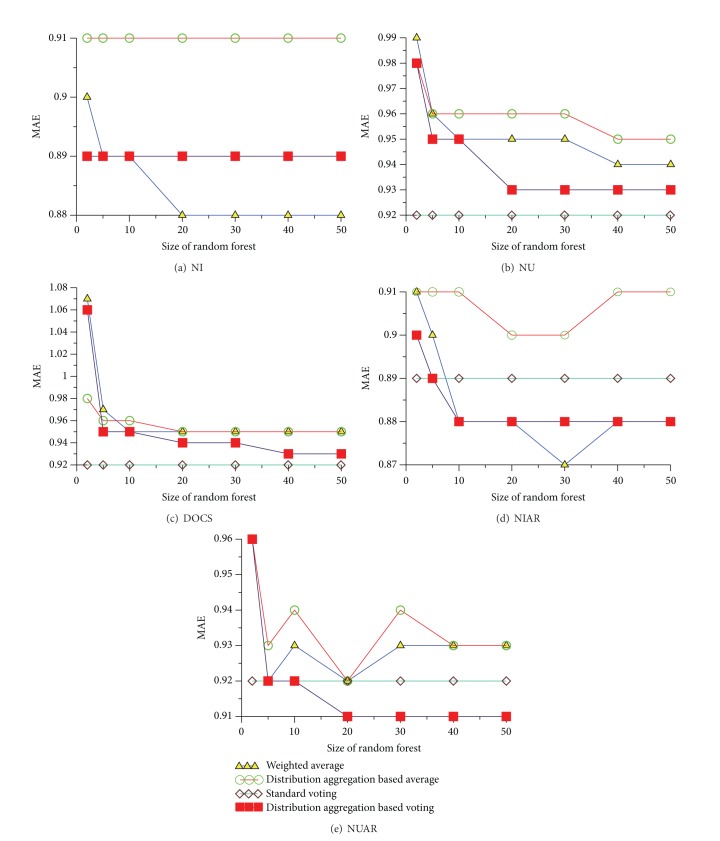
Comparisons of different prediction approaches.

Figure 4(a) compares four approaches of NI. MAE's range of four predicting approaches is between 0.88 and 0.91. Weighted average approach is the best in four ones. The precision of weighted average keeps stable when the size of the forest is greater than or equal to 20. However, the precision of three other approaches has kept stable.

Figure 4(b) compares four approaches of NU. MAE's range of four predicting approaches is between 0.92 and 0.99. Standard voting approach is the best in four ones. The precision of standard voting has kept stable. However, the precision of three other approaches keeps stable when the size of the forest reaches a certain value.

Figure 4(c) compares four approaches of DOCS. MAE's range of four predicting approaches is between 0.92 and 1.07. Standard voting approach is the best in four ones. The precision of standard voting has kept stable. However, the precision of three other approaches keeps stable when the size of the forest reaches a certain value.

Figure 4(d) compares four approaches of NIAR. MAE's range of four predicting approaches is between 0.88 and 0.91. Weighted average approach is the best in four ones. The precision of standard voting has kept stable. However, the precision of three other approaches keeps stable when the size of the forest reaches a certain value.

Figure 4(e) compares four approaches of NUAR. MAE's range of four predicting approaches is between 0.91 and 0.96. Distribution aggregation based voting approach is the best in four ones. The precision of standard voting has kept stable. However, the precision of three other approaches keeps stable when the size of the forest reaches a certain value.

In summary, we know from Figure 4 that (1) the precision in terms of MAE is stable on the whole when size of random forest is 2 to 20. Among these approaches, the precision of standard voting has kept stable; (2) NI approach is more precise than NU one. NU approach is more precise than DOCS one; (3) aggregated-based algorithms with AR are more slightly precise than without one. NIAR approach is almost the same as NI. The two approaches are the most precise in all prediction ones. One reason is that it is based on user rating history which forms his/her preference. They yield a MAE of 0.88 (on a five-point rating scale) on movie rating datasets.

## 5. Discussions

To the best of the authors' knowledge, little is known in previously published studies of aggregate in recommender systems. The work is related to previously published works on model-based RSs and group recommender.

Model-based RSs use the demographic, item information, and collection of ratings to create a model that generates the recommendations [8]. Model-building methods work by creating a model offline and then running the model online. Among the most widely used models, there are Bayesian classifiers [6], neural networks [22], and decision tree [23]. These models have been used to solve three kinds of cold-start problems [8]: new community, new item, and new user. The new community problem refers to the difficulty in obtaining a sufficient amount of data (ratings) for making reliable recommendations. The new item problem [8] arises because the new items entered in RS do not usually have initial ratings. The new user problem represents one of the great difficulties faced by the RS in operation. Since new user has not yet provided any rating in the RS, he/she cannot receive any personalized recommendations based on memory-based RS.

The random forest [13] is composed of many decision trees. Decision tree is a general computational model represented as a set of if-then rules [24]. Each tree is built based on a different set of training data and grown to the largest extent possible without pruning. Each splitting or decision node is acted by the best splitting attribute from randomly selected subset of the conditional attributes. To classify a new object, each tree in the forest gives a classification. The final classification of the object is determined by majority of votes among the classes decided by the forest of trees.

As defined in [10, 25], there are two strategies commonly adopted for generating group recommendations: the aggregated models and aggregated predictions. The former combines individual user models, that is, individual user profiles that capture the preferences of a group member into a group model from where items are recommended for the group are identified, whereas the latter generates predictions for individual group members and then aggregates the predictions to suggest items for the group. In this paper we present our proposed aggregated recommender, which predicts the rating of a group of users to a kind of items.

## 6. Conclusions

In this paper, we proposed random forest approaches to aggregated recommendation. By comparing several predicting approaches, we may draw the following conclusions: (1) MAE is stable on the whole when size of random forest is 5 to 20; (2) aggregated recommender can be used to solve NI, NU, and DOCS problems; (3) the precision of the NI approach is the highest; (4) the attribute of AR can improve the predicting accuracy; (5) the precision of DOCS approach maintains an acceptable level.

## Figures and Tables

**Algorithm 1:**
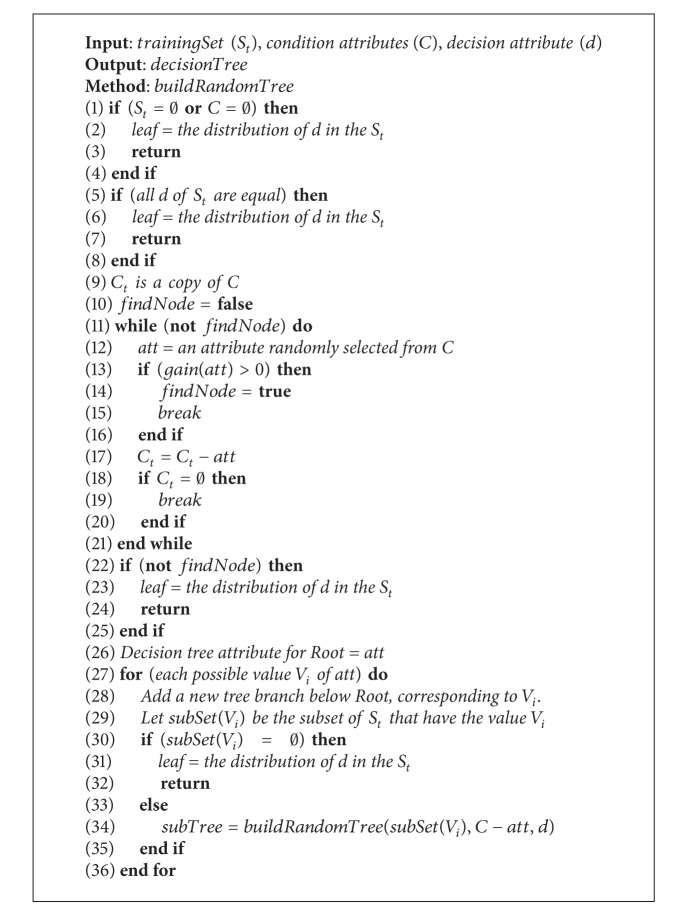
Random decision tree.

**Algorithm 2:**
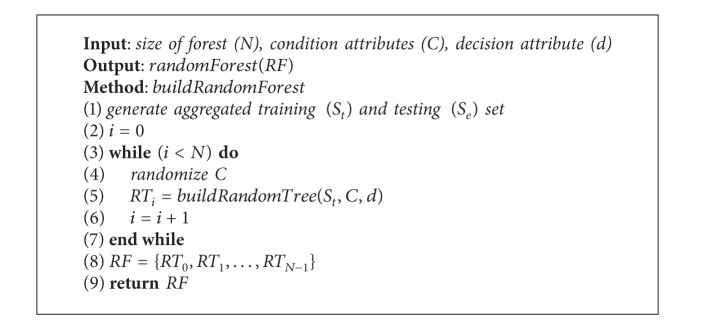
Random forest.
